# Characterization of a nontypeable *Haemophilus influenzae* thermonuclease

**DOI:** 10.1371/journal.pone.0197010

**Published:** 2018-05-10

**Authors:** Christine Cho, Aroon T. Chande, Lokesh Gakhar, Jason Hunt, Margaret R. Ketterer, Michael A. Apicella

**Affiliations:** 1 Iowa Inflammation Program, The University of Iowa, Iowa City, Iowa City, IA, United States of America; 2 Department of Infectious Disease, University of Iowa Hospitals, Iowa City, IA, United States of America; 3 Physician Scientist Training Pathway, University of Iowa Carver College of Medicine, Iowa City, IA, United States of America; 4 School of Biological Sciences, Georgia Institute of Technology, Atlanta, Georgia, United States of America; 5 IHRC-Georgia Tech Applied Bioinformatics Laboratory, Atlanta, Georgia, United States of America; 6 PanAmerican Bioinformatics Institute, Cali, Valle del Cauca, Columbia; 7 Department of Biochemistry, The University of Iowa, Iowa City, IA, United States of America; 8 Protein Crystallography Facility, The University of Iowa, Iowa City, IA, United States of America; 9 Institute for Environmental Studies, Western Illinois University, Macomb, IL, United States of America; 10 Department of Microbiology, The University of Iowa, Iowa City, IA, United States of America; Emory University School of Medicine, UNITED STATES

## Abstract

Nontypeable *Haemophilus influenzae* (NT*Hi*) has been shown to form biofilms, comprised of extracellular DNA (eDNA), in the middle ear and bronchus during clinical infections. Studies in our laboratory have shown that NT*Hi* possesses a homolog of *Staphylococcus aureus* thermonuclease (staphylococcal thermonuclease), NT*Hi* nuclease (NT*Hi* Nuc, HI_1296). This enzyme had similar size, heat stability, and divalent cation requirements to those of the staphylococcal homolog as determined by light scattering and circular dichroism spectroscopy. Small angle X-ray scattering (SAXS) analysis suggested an overall shape and substrate-binding site comparable to those of staphylococcal nuclease. However, NT*Hi* Nuc was approximately 25-fold more active in fluorescence resonance energy transfer (FRET) activity assay than staphylococcal thermonuclease. Homology modeling implicates shorter NT*Hi* Nuc loops near the active site for this enhanced activity.

## Introduction

Nontypeable *Haemophilus influenzae* (NT*Hi*) are frequently found as a member of the normal upper respiratory tract bacterial flora. This species can be a frequent cause of airway infections, including otitis media in children, and sinusitis and acute exacerbations of chronic bronchitis in adults [1]. NT*Hi* has been shown to be capable of forming biofilms *in vitro* and in the upper and lower human respiratory tract during human disease [2–4]. Bacterial biofilm matrix is an elaborate network of molecules, which can include pili, polysaccharides, double stranded extracellular DNA (eDNA), and bacterial and host derived substances that help shape and secure the biofilm to an inanimate or host surface [5]. Recent studies have shown that *Pseudomonas aeruginosa* in cystic fibrosis lung disease forms aggregates of microbes containing dead and dying neutrophil, and appear to be active similar to stationary biofilms [6, 7]. The matrix protects the underlying bacteria from assault by the host immune response and antibiotic treatment thus contributing to the recalcitrant nature of biofilm to treatment [8]. An open reading frame with high homology to staphylococcal thermonuclease, called NT*Hi* nuclease (NT*Hi* Nuc, HI_1296), is present in the sequenced genome of *H*. *influenzae* strains 2019, KW20 Rd and 86-028NP. We have previously found that this nuclease is an important factor in remodeling NT*Hi* biofilm [9].

In this study, we present biophysical and functional characterization of NT*Hi* Nuc. FRET based enzyme kinetic studies suggested that NT*Hi* Nuc was ~25 times more active than staphylococcal thermonuclease. SAXS analysis indicated that NT*Hi* Nuc had a structure and DNA binding cleft similar to those of staphylococcal thermonuclease. Like the staphylococcal thermonuclease, this enzyme retained activity after heating to 65°C and required a divalent cation, magnesium or calcium, for optimal activity. This stability and increased activity of NT*Hi* Nuc could play a role in efficacy of NT*Hi* biofilm remodeling.

## Materials and methods

### Bacteria and culture conditions

*Escherichia coli* (*E*. *coli*) BL21*(DE3) was grown in Luria-Bertani (LB) medium with or without agar and supplemented with IPTG as needed.

### Cloning, expression and purification of NT*Hi* Nuc

NT*Hi* Nuc without signal sequence was expressed with a cleavable 6x His-tag in pET151/D-TOPO (Life Technologies Corporations) in BL21* (DE3) *E*. *coli* cells and induced with 1.5 mM IPTG (Invitrogen) at 18°C. Cell cultures were pelleted and frozen at -20°C in preparation for lysis. The frozen cells were resuspended in 100 mM Tris, pH 9.1, 5 mM CaCl_2_, 200 mM NaCl, 50 mM imidazole (lysis buffer) plus a mini cOmplete protease inhibitor cocktail tablet (Roche) then homogenized with an Emulsiflex 2000. His-tagged NT*Hi* Nuc was bound to Ni-NTA affinity resin (Qiagen) and washed with the lysis buffer then eluted with elution buffer (lysis buffer with 250 mM imidazole). The eluted protein was concentrated and mixed with lab-purified Tobacco Etch Virus protease at 1:200 in 4°C for 16 hours to cleave the His-tag. The cleaved products were passed over a Ni Sepharose column (HisTrap FF, GE Healthcare) and the flow-through was loaded onto a gel filtration column (Superdex 75 25/60, GE Healthcare) to separate NT*Hi* Nuc from contaminants and to exchange buffer into the final purification and reaction buffer: 100 mM Tris, pH 9.1, 5 mM CaCl_2_, 200 mM NaCl, 5 mM DTT.

### FRET assay

Nuclease enzyme activity was measured by a fluorescence resonance energy transfer activity study (FRET) assay using a Tecan Infinite M200 Pro. The FRET substrate was a single-stranded 30-mer oligonucleotide (Cy3-CCGCGAGAAACCAAGCACAGAGCACCGAAGA-BHQ_2) with the 5’ end modified with Cy3 fluorophore and the 3’ end modified with Black Hole Quencher 2 [10]. The substrate was incubated with either NT*Hi* Nuc, *S*. *aureus* thermonuclease (Sigma-Aldrich) or RNase-free bovine pancreatic DNase I (New England Bioscience, positive control), both enzymes in DNase I reaction buffer (New England Bioscience), at 26°C, and then the absolute fluorescence was measured every 10 s for 5 min. Relative activity was calculated by,

$$relative\;activity=\frac{v/nM}{v_{d}/nM_{d}}$$

*v* is the reaction velocity of the enzyme of interest and *nM* is the concentration (nM) of the enzyme of interest. *v_d_* is the reaction velocity of staphylococcal thermonuclease and *nM_d_* is the staphylococcal thermonuclease concentration (nM). *v*/*nM* of pancreatic DNaseI was calculated based on published data [11].

Each study shown was performed a minimum of six times on multiple NT*Hi* Nuc samples.

### Dynamic and static light scattering

A DynaPro NanoStar instrument (Wyatt Technology, Santa Barbara) was used for all light scattering experiments and data were collected and processed with Dynamics v7 (v.7.1.7.16). Purified NT*Hi* Nuc was filtered through a 0.02 μm Whatman Anotop filter and the final concentration was measured by Nanodrop 200. A 2 μl quartz cuvette was used for static light scattering (SLS), while disposable 50 μl Eppendorf UVettes (Fisher catalog # 952010051) and 4 μl plastic cuvettes (Wyatt Technology catalog # 162960 rev C) were used for dynamic light scattering (DLS). SLS measured the absolute average intensity of scattering, and DLS measured the fluctuation around the average intensity. Through multiple runs of static light scattering (SLS) and dynamic light scattering (DLS), we determined hydrodynamic radius, polydispersity, and calculated molecular weight with standard deviations.

### Melting point analysis

The DynaPro NanoStar in DLS mode was used to conduct melting temperature experiments in both 50 μl and 4 μl plastic cuvettes on filtered NT*Hi* Nuc samples. Rubber caps were used to seal the sample chambers during temperature ramps to minimize sample evaporation. Scattering intensity and hydrodynamic radius data were recorded as temperature was ramped at 1.0°C/min from 25°C until the protein unfolded. A sudden rapid increase of these parameters from the baseline marks the onset of protein unfolding. These T_onset_ values with standard deviations were calculated using the linear fit analysis module in Dynamics v7. Melting point data was validated with Jasco J-815 Circular Dichroism Spectropolarimeter (21-Q-1, Starna Cells Inc.) [12]. Temperature was increased by 1°C/min and spectra collected from 260–190 nm with data pitch of 1 nm, bandwidth of 1 nm, data integration time of 2 sec and scanning speed of 100 nm/min. Circular Dichorism (CD) spectrum measurements were also taken at fixed temperatures using the same parameters but with 10 measurements/spectra/temperature for higher accuracy. Purified NT*Hi* Nuc was diluted to 0.5 mg/ml in phosphate buffer (PBS + 0.003% Tween-20) to reduce buffer background in CD experiments.

### Homology modeling

A homology model of NT*Hi* Nuc was generated using the hm_model macro in the program YASARA (YASARA Biosciences). High-resolution (1.5–1.9 Å) crystal structures of staphylococcal nuclease were used as templates and the best of five models (in terms of dihedral angles and packing scores) after simulated annealing minimization was selected as the homology model.

### Small-angle X-ray scattering (SAXS)

Purified NT*Hi* Nuc was dialized against SAXS buffer (100 mM Tris, pH 7 or 9, 150 mM NaCl, 5 mM CaCl, 5 mM DTT). SAXS data were collected at the SIBYLS beamline (beamline 12.3.1) at the Advanced Light Source, Lawrence Berkeley National Laboratory. The data were collected at 10°C at three concentrations in the range of 1.2–3.7 mg/ml for the sample at pH 7 and 0.4–1.2 mg/ml for the sample at pH 9. The samples were progressively exposed to radiation for 0.5, 1, 2 and 4 seconds. Radiation damage was observed for the highest exposure for all samples and those data were not used for further analysis. Scattering data from the dialysate buffer were collected and subtracted from the protein solution scattering data. No concentration-dependent scattering was noted based on data processed from three different concentrations. Data processing was performed using the PRIMUS program package [13]. Analysis of the Guinier region (low-q region) of the scattering data for all samples at all concentrations and exposures (except the highest) was linear, suggesting that the proteins did not undergo aggregation or interparticle interference. The radius of gyration, R_g_, was evaluated using the Guinier approximation at low q where qR_g_ < 1.3 [14]. The program GNOM [15] was used to compute the pair distribution function, P(r), and the maximum dimension of the macromolecule, D_max_. Experimental SAXS data were compared with simulated scattering curves in the program CRYSOL [16] for the homology model of NT*Hi* Nuc and the crystal structure of *Staphylococcal* thermonuclease (pdb id 1STN). *Ab initio* molecular envelopes were generated from the experimental scattering data using both DAMMIF [17] and GASBOR [18]. Only the highest concentration and highest exposure scattering data without radiation damage were used for *ab initio* reconstructions. Eight molecular envelopes were obtained using identical parameters but different randomly chosen values to seed the calculation. The coordinates for the models from eight runs were superimposed, averaged and filtered in the programs SUPCOMB [19] and DAMAVER [20] to create an average model, which represented the general structural features of each reconstruction, and a filtered model, which represented the core conserved features of all reconstructions. The derived molecular envelopes were visualized in PyMOL (v.1.6; Schrödinger LLC).

### Statistical analysis

Statistical analysis was performed with Prism 6 software (GraphPad Software, Inc., La Jolla, CA). Values that met a *p* value cutoff of 0.05 were considered statistically significant.

## Results

### NT*Hi* Nuc (HI_1296) is homologous to staphylococcal thermonuclease

**Fig 1:**
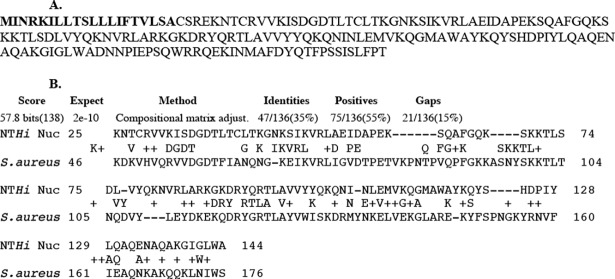
Amino acid sequence of NT*Hi* Nuc and its homology to staphylococcal thermonuclease. Fig 1A shows the amino acid sequence of NT*Hi* 2019 *Nuc* (HI_1296). The first 20 amino acids in bold are the signal sequence of the protein. Fig 1B shows the alignment of NT*Hi Nuc* with staphylococcal thermonuclease, which has 35% conserved amino acids and Expect value (number of chance matches) of 2e-10, indicating a significant match.

Complete amino acid sequence of NT*Hi* 2019 *Nuc* is shown in Fig 1A with the signal sequence highlighted. Fig 1B shows that NT*Hi Nuc* is homologous to staphylococcal thermonuclease with 35% conserved amino acids and E-value of 2e-10.

#### Enzymatic and physicochemical characterization of NT*Hi* nuclease

**Fig 2:**
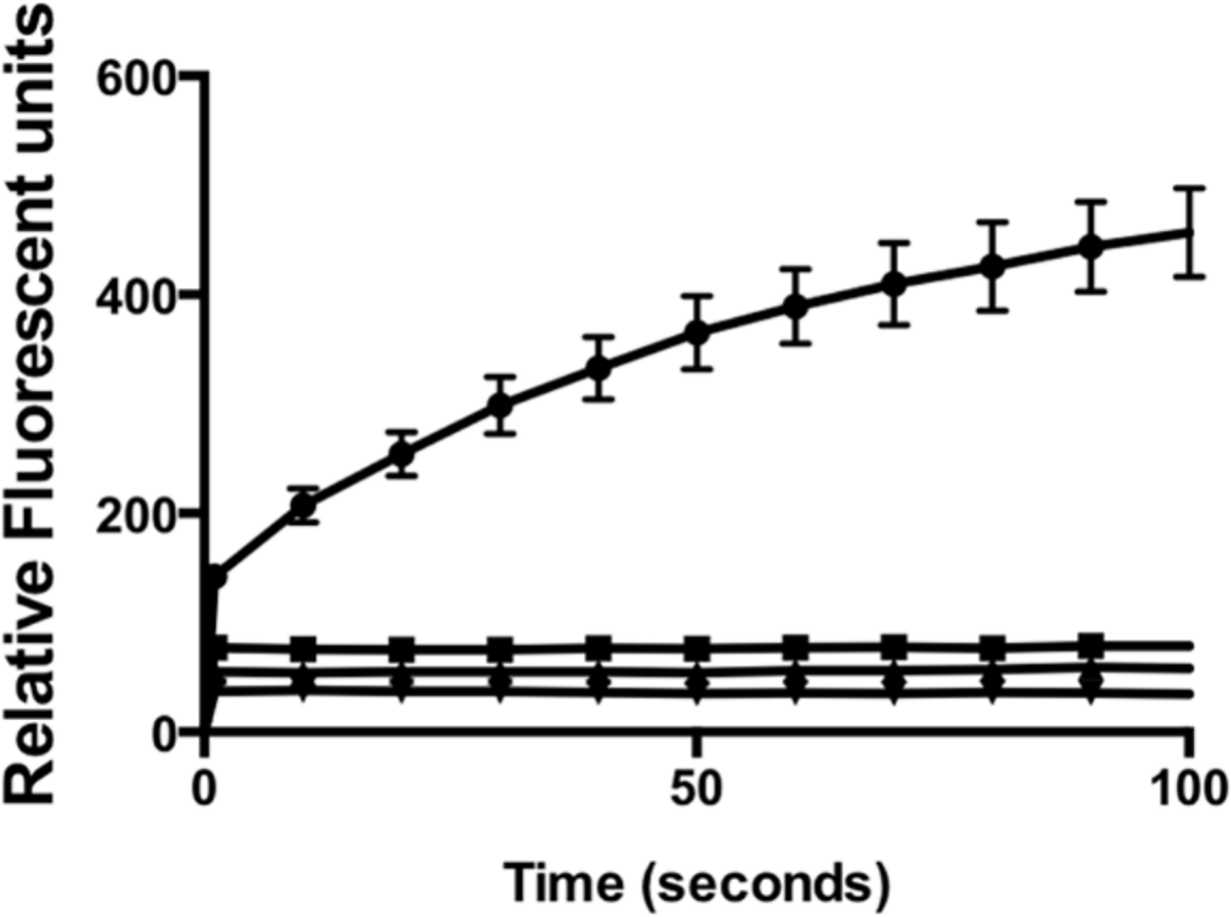
Effect of EDTA on NT*Hi* Nuc activity. FRET assay shows single-stranded nuclease activity of NT*Hi* Nuc activity. NTHi Nuc requires divalent cation as it lost activity with addition of EDTA. The respective lines are 0.03 nM NT*Hi* Nuc alone (●), 0.03 nM NT*Hi* Nuc and 0.3nM EDTA (■), 0.3nM EDTA alone (▲) and distilled water (▼). The experiment was repeated three times.

**Fig 3:**
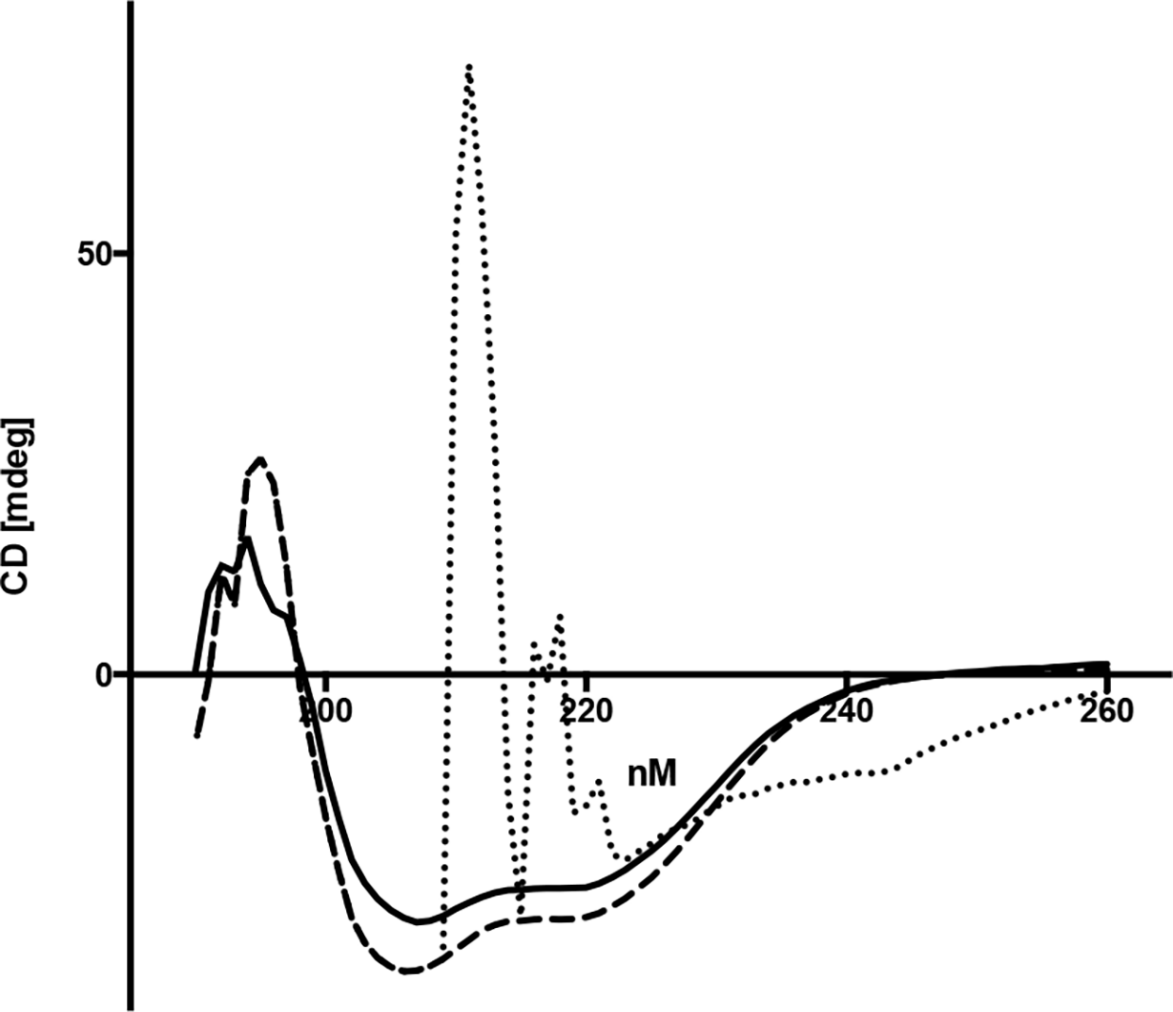
CD spectra of NT*Hi* Nuc from 190–260 nm. This figure shows representative curves. When NT*Hi* Nuc was heated from 25°C (solid line) to 60°C (dotted line), the spectra was lost, indicating loss of secondary structure. When the sample was then cooled to 25°C (dashed line), NT*Hi* Nuc underwent reversible thermal folding. Samples were referenced to buffer.

**Fig 4:**
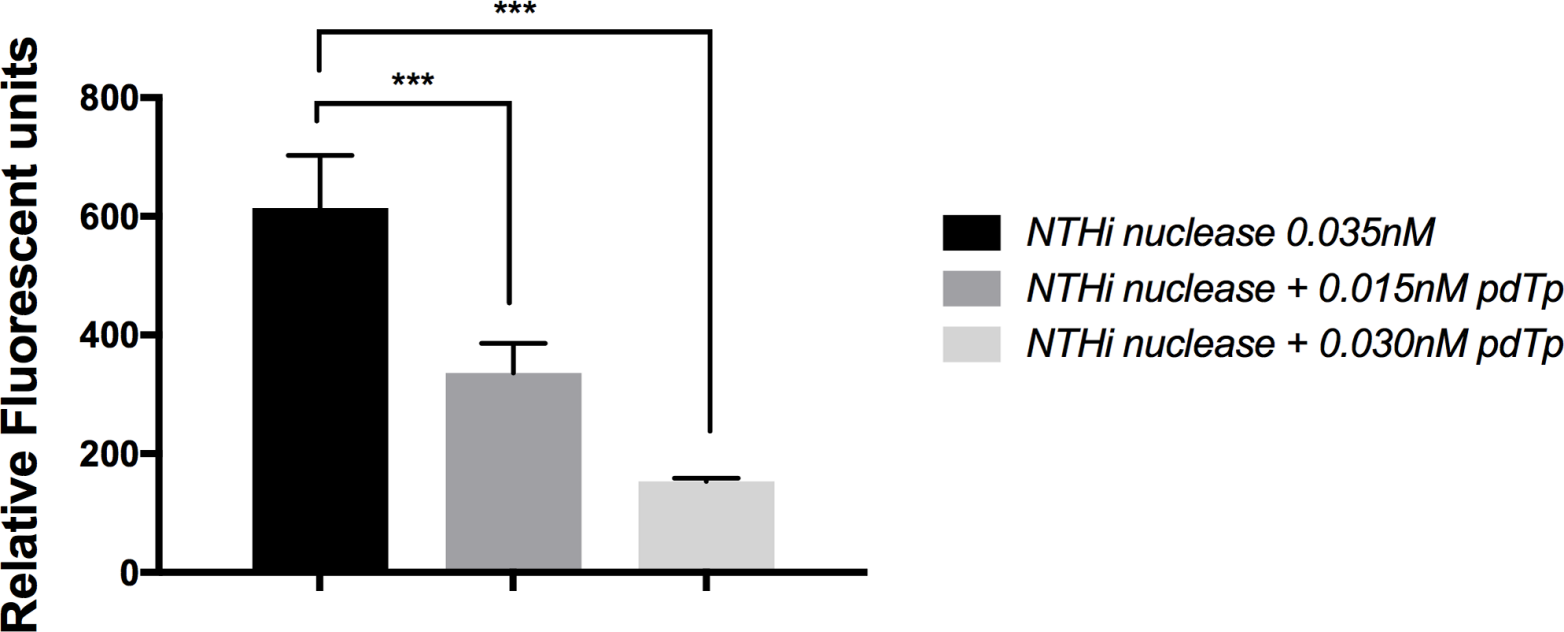
Effect of inhibitor on NT*Hi* Nuc activity. NT*Hi* Nuc was incubated with 2 μM FRET substrate and increasing concentrations of inhibitor (pdTp). Enzyme activity was measured in FRET assay. n = 3 for 0.03nM of pdTp and n = 2 for 0.015nM of pdTp. Relative fluorescent units decreased as pdTp concentration increased, thus indicating that the inhibitor acts as a competitive inhibitor for NT*Hi* Nuc when low concentration of the substrate was present, as measured by FRET. Bars represent the average from three independent experiments for 0.03nM of pdTp and two independent experiments for 0.015nM of pdTp ± SD. p-values were determined using two-sided student’s t test with assumption of equal variance (*** p-value < 0.0005).

Enzyme activity studies were performed with PCR grade staphylococcal thermonuclease (Sigma-Aldrich) and highly purified recombinant NT*Hi* Nuc without signal sequence. Signal sequence was determined by LipoP 1.0 analysis. NT*Hi* Nuc possessed both single-stranded (Fig 2) and double-stranded DNA [9] nuclease activity. NT*Hi* Nuc was a highly efficient nuclease with greater than 1500 fold increased activity compared to bovine pancreatic DNase I and greater than 25 fold increased activity compared to staphylococcal thermonuclease (Table 1). NT*Hi* Nuc activity was greatest near pH 9, similar to staphylococcal thermonuclease, however NT*Hi* Nuc retained some activity over a range of pH 5–9.5 (data not shown). NT*Hi* Nuc activity was also temperature dependent with maximum at 37°C. Of note, NT*Hi* Nuc remained active at 4°C and 25°C for several months in sterile solution and retained endo-exonuclease activity above 45°C in pH = 8.5; heating to 60°C partially denatured the protein. Heat-denatured NT*Hi* Nuc, when cooled to 25°C, re-established its activity and was able to degrade DNA (Fig 3). NT*Hi* Nuc and staphylococcal thermonucleases are most active near their pIs [21], similarly both staphylococcal thermonuclease and NT*Hi* Nuc are competitively inhibited by thymidine 3,5-bisphosphate (pdTp), a DNA mimic [22]. Half and equimolar concentrations of pdTp impacted initial rates of endonuclease activity (Table 1). However, at low substrate concentration at 300 seconds, half-molar nuclease-pdTp reactions had approximately twice the activity of equimolar nuclease-pdTp reactions (Fig 4).

**Table 1:** FRET assay of NT*Hi* Nuc activity.

Sample	Concentration nM	Velocity/nM	Relative activity compared to staphylococcal thermonuclease
NT*Hi* Nuc	0.035	158.4	23
NT*Hi* Nuc + 0.015 nM pdTp	0.035	26.3	4
NT*Hi* Nuc + 0.03 nM pdTp	0.035	20.3	3
Staphylococcal thermonuclease	0.278	6.8	1
Bovine pancreatic DNase I	6	0.1*	0.015

* calculated based on published data [11]

### Divalent cation dependence

**Fig 5:**
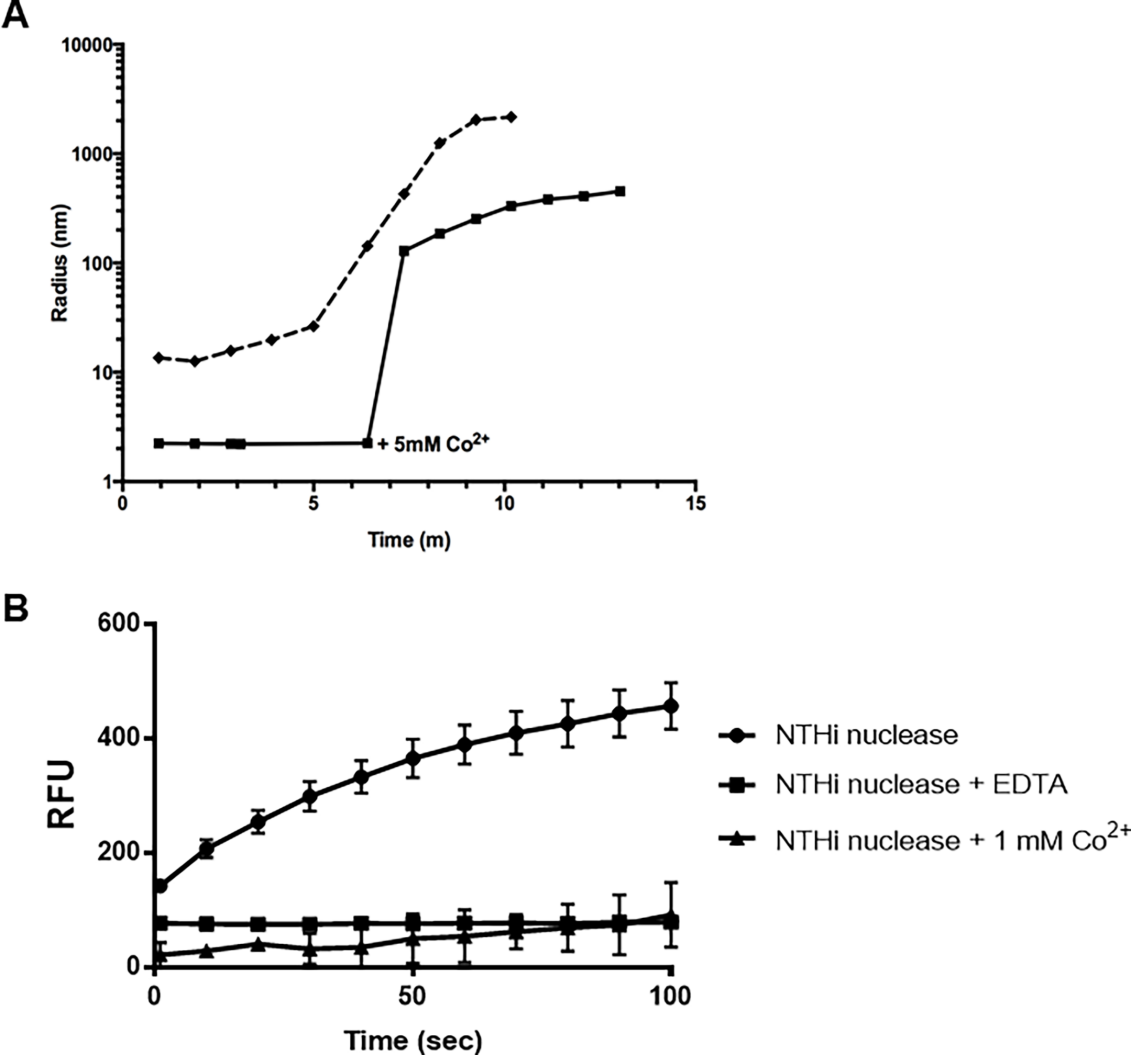
Effect of Co^2+^ on NT*Hi* Nuc structure and activity. NT*Hi* Nuc with Ca^2+^ has a hydrodynamic radius of approximately 2.1 nm when in solution. Within minutes of adding Co^2+^, a known inhibitor for staphylococcal thermonuclease, the size of NT*Hi* Nuc increased dramatically indicating oligomerization or nonspecific aggregation (solid line in Panel A). The aggregates formed by the addition of Co^2+^ to NT*Hi* Nuc were briefly disrupted by passing the solution through a 0.22 μm membrane filter reduced the hydrodynamic radius to approximately 15 nm (start of dashed line in Panel A). However, NT*Hi* Nuc continued aggregating and increasing in size due to Co^2+^ still present in solution (dashed line). Panel A shows representative curves. Activity of NT*Hi* Nuc was quenched with addition of Co^2+^ when measured by FRET, similar to when EDTA was added (Panel B). Panel A shows representative curves and Panel B was done in triplicates.

EDTA chelation quenched NT*Hi* Nuc activity (Fig 2), highlighting divalent cation requirement for its activity. Addition of Ca^2+^ or Mg^2+^ restored NT*Hi* Nuc activity (data not shown). Either Ca^2+^ or Mg^2+^ was required, but not both, as NT*Hi* Nuc activity was unchanged when both Ca^2+^ or Mg^2+^ were added (data not shown). Cobalt, an inhibitor of staphylococcal thermonuclease [23], not only aggregated NTHi Nuc structure (Fig 5. Panel A), but also quenched its activity, similar to addition of EDTA (Fig 5. Panel B).

### Static and dynamic light scattering

Static and Dynamic Light Scattering (SLS/DLS) were used to determine the biophysical characteristics of NT*Hi* nuclease. Molecular weight determination from SLS shows no oligomerization at pH 7 (Table 2). At pH 9, hydrodynamic radius increased, indicating a larger open conformation (Table 2). Protein stability at various pH was reported as melting temperatures, T_onset,_ in Table 3. NT*Hi* Nuc was most stable around pH 9 with a reported T_onset_ of 53 °C. Comparatively, the T_onset_ of staphylococcal thermonuclease is 45.8 °C at pH 7 (calculated from published data; [24]). At pH 7, excess pdTp increased NT*Hi* Nuc thermal stability, reported as a T_onset_, from 40 °C to 48 °C (Table 3). In addition to pH dependence, NaCl and divalent cations were assayed for effects on stability of NT*Hi* Nuc. DLS showed that NT*Hi* Nuc was stable between 50 mM and 1M of NaCl, below and above physiological concentrations (data not shown). When Ca^2+^ was replaced with Co^2+^, NT*Hi* Nuc aggregated (Fig 5. Panel A) and activity was lost (Fig 5. Panel B). Passing NT*Hi* Nuc + Co^2+^ through a 0.22 μm membrane filter removed these aggregates and re-aggregation can be quantified in real time using DLS (Fig 5. Panel A).

**Table 2:** Light Scattering demonstrating effect of pH on NT*Hi* Nuc size.

pH	Radius (nm)	Polydispersity (%)	SLS MW (kDa)
7	2.19 ± 0.04	13.1 ± 0.3	19.3 ± 0.0
8	2.64 ± 0.04	32.5 ± 1.8	—
9	2.53 ± 0.01	20.3 ± 1.1	—

**Table 3:** Effect of pH on thermal stability.

	T_onset_
NT*Hi* Nuc pH 7	40.2 ± 0.5
NT*Hi* Nuc pH 7+ pdTp (excess)	48.1 ± 1.45
NT*Hi* Nuc pH 8	45.1 ± 0.1
NT*Hi* Nuc pH 9	53.2 ± 0.1
Staphylococcal thermonuclease pH 7^1^	45.8
Staphylococcal thermonuclease pH 8^2^	46

^1^ –Calculated from published data [24, 25]

^2^ –Calculated from published data (38)

### Small-angle X-ray scattering

**Fig 6:**
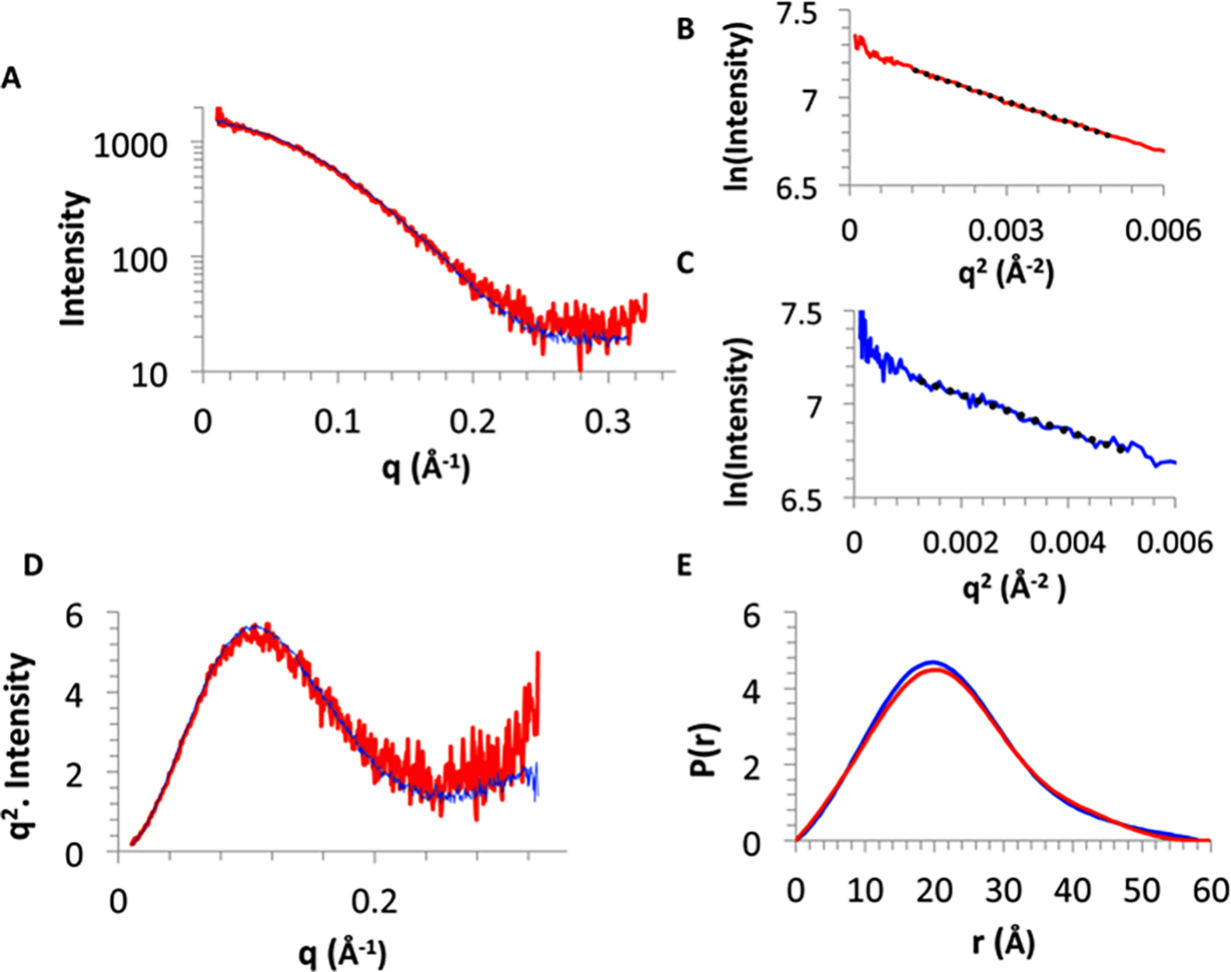
SAXS analysis of NT*Hi* Nuc. Fig 6A shows scaled SAXS scattering curves of NT*Hi* Nuc in pH = 7 (blue) and 9 (red). There were no gross structural changes at those two pH. Fig 6B and Fig 6C show Guinier plot for NT*Hi* Nuc at pH = 7 and 9, respectively. Rg was 17.4 Å at both pH. Fig 6D shows parabolic Kratky plots in pH 7 (blue) and 9 (red), showing some flexible region. Fig 6E shows pairwise distribution function plots at pH 7 (blue) and 9 (red), with maximum dimensions of 58.3 Å at pH 7 and 59.6 Å at pH 9.

**Fig 7:**
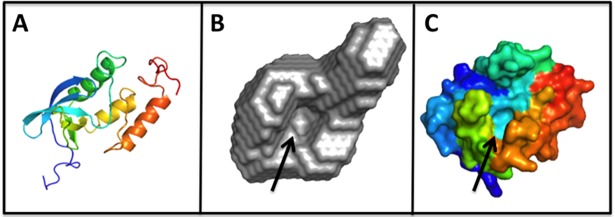
Structure of NT*Hi* Nuc by SAXS. Fig 7A shows a homology model of NT*Hi* Nuc shown as a cartoon, blue = N-terminal and red = C-terminal. Fig 7B shows the *ab initio* shape of NT*Hi* Nuc determined from SAXS data at pH 7. Fig 7C shows the surface representation of staphylococcal thermonuclease crystal structure. Fig 7B and 7C show similar overall shape and active site (black arrow).

**Fig 8:**
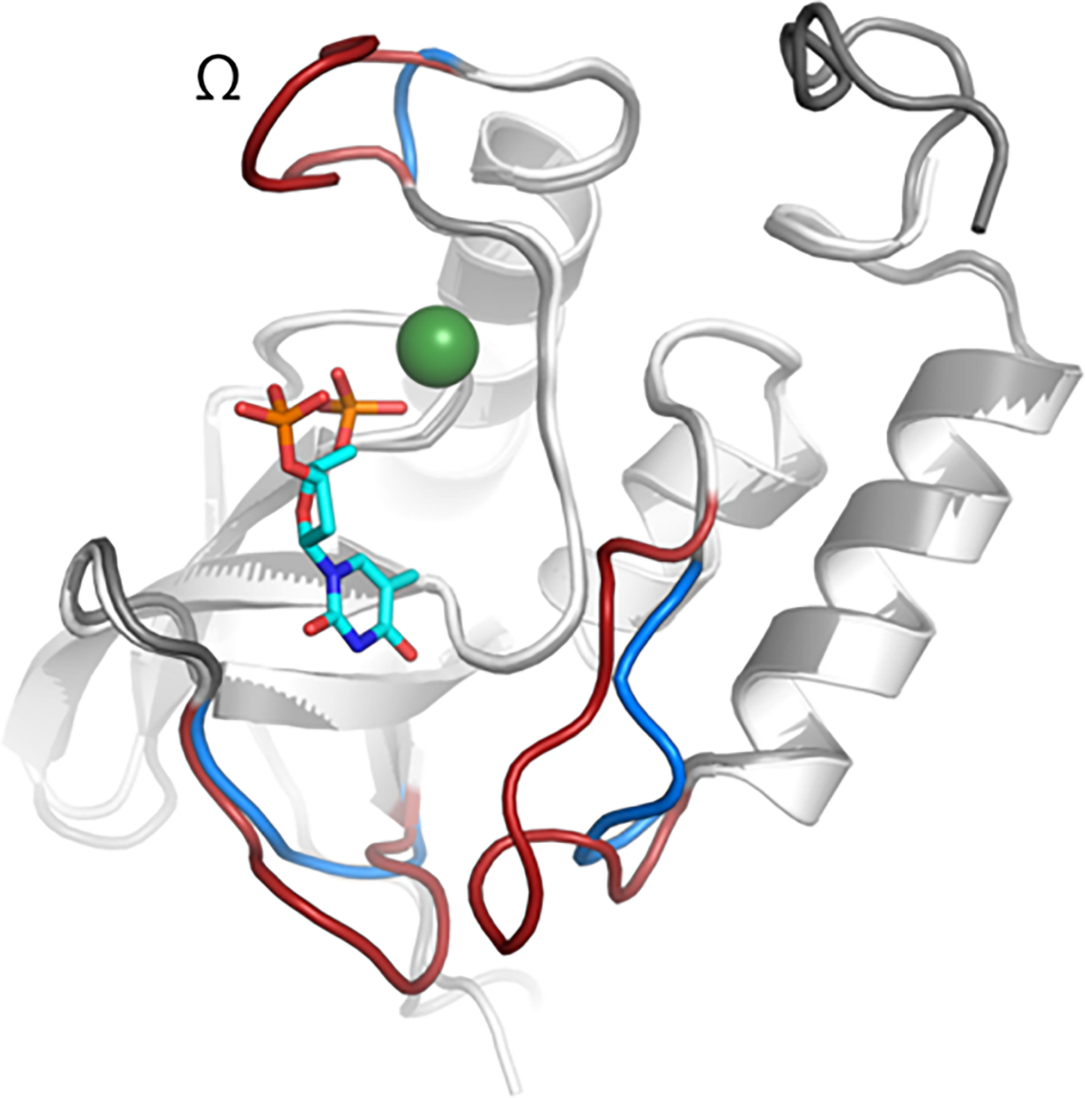
Homology model comparison of NT*Hi* Nuc to staphylococcal thermonuclease. Overlay of the homology model of NT*Hi* Nuc (blue), and the active site of staphylococcal thermonuclease (pdb id 1STN) (red). Green sphere represents calcium and cyan sticks represent TdtP (inhibitor); modeled from pdb id 2SNS. There are three loops in the active site of NT*Hi* Nuc that are shorter than those of staphylococcal thermonculease. The absence of Ω loop near the active site of NT*Hi* Nuc compared to the staphylococcal thermonuclease could contribute to the stronger activity of NT*Hi* Nuc.

The Guinier analysis resulted in R_g_ values of 17.4 Å at both pH 7 and pH 9. The radius of gyration of the homology model as calculated by CRYSOL was 17.0Å indicating that NT*Hi* Nuc was a monomer in solution. Pairwise distance distribution function, P(r), showed maximum dimension, D_max_, of 58.3 and 59.6 Å (Fig 6) at pH 7 and 9, respectively. These data indicate that despite the significant difference in activity of NT*Hi* Nuc at pH 7 and 9, there were no global conformation changes. The *ab initio* model had features, such as the active site cavity, that were remarkably similar to that of staphylococcal nuclease (Fig 7) indicating similarity between NT*Hi* nuclease and staphylococcal thermonuclease. However, the three loops, including the Ω loop near the calcium ion, forming the metal binding and active site in NT*Hi* Nuc homology model were predicted to be shorter than the corresponding loops in staphylococcal nuclease (Fig 8).

## Discussion

A number of bacterial species responsible for causing human disease including *Staphylococcus aureus*, *Neisseria gonorrhoeae*, *Streptococcus sanguinis*, *Serratia marscens*, *Campylobacter jejuni*, *Enterococcus faecalis* and *Bacillus anthracis* encode a thermonuclease. The functional properties and structure of the *S*. *aureus* thermonuclease has been studied for almost 50 years [26]. Studies have shown that *S*. *aureus* and *N*. *gonorrhoeae* encode nucleases, which play a role in remodeling the extracellular DNA (eDNA) matrix of the biofilm formed by the respective organisms [27, 28]. In addition, it has been shown that the nucleases of *N*. *gonorrhoeae*, *S*. *aureus and S*. *sanguinis* protect the microbes from neutrophil extacellular trap-mediated bactericidal activity [29–31]. Our previous study evaluated the role of NT*Hi* Nuc in biofilm by NT*Hi* [9]. This study further characterizes the NT*Hi* enzyme in detail. NT*Hi* Nuc has 35% identity and 55% similarity to staphylococcal thermonuclease. Similar to the staphylococcal thermonuclease, NT*Hi* Nuc could be inactivated by heating to 65 °C, but could refold to its active form at 25°C. Unlike staphylococcal nuclease, which has no cysteines, NT*Hi* Nuc has three cysteines, two of which were predicted to be on neighboring beta strands in the homology model, capable of forming a disulfide bond that could potentially contribute to its high thermostability. NT*Hi* Nuc was approximately 25 and 1500 times more potent than staphylococcal thermonuclease and DNase I, respectively. Low resolution SAXS analysis indicated that NT*Hi* Nuc had a similar binding pocket and general shape as staphylococcal thermonuclease, but predicted to have shorter loops forming the metal binding and active sites compared to those of staphylococcal thermonuclease, including the Ω loop. Previous studies have shown that the deletion of staphylococcal thermonuclease Ω loop had increased stability compared to the staphylococcal thermonuclease with an active site mutation [32, 33]. Therefore, the absence of the Ω loop in NT*Hi* Nuc could play a role in the enhanced activity of the NT*Hi* Nuc. The other two shorter loops in NT*Hi* Nuc active site could also allow space for larger substrates, which may also contribute to the enhanced activity of the NT*Hi* Nuc. The melting temperatures and optimal pH for enzymatic activity are similar for both enzymes. NT*Hi* Nuc required divalent cations, Mg^2+^ or Ca^2+^, for its activity. NT*Hi* Nuc can digest both double- and single-stranded eDNA.

Our previous work has shown that NT*Hi nuc* expression is crucial for biofilm remodeling and dispersal. We were surprised that while the nuclease was regulated, there was only 1.52-fold increased gene expression in planktonic phase compared to those of biofilm phase. The high potency of NT*Hi* Nuc suggests that this tight regulation of expression was necessary to disperse organisms from biofilm and was required to maintain biofilm homeostasis.

The use of exogenous nucleases has been suggested to treat chronic biofilm infections in the respiratory tract in illnesses such as cystic fibrosis [34, 35]. Given the potency of the NT*Hi* nuclease, it may find a role in management of such disease processes. In addition, the NT*Hi* nuclease could find usefulness in industrial processes such as production of flavor enhancers [36] and in treatment of food processing equipment contaminated with DNA containing biofilms [37].
